# Spontaneous intracranial hypotension associated with cerebral venous thrombosis detected by a sudden seizure: a case report

**DOI:** 10.1186/s40981-020-00362-3

**Published:** 2020-08-04

**Authors:** Atsuko Yamamoto, Yoshiyasu Hattammaru, Shoichi Uezono

**Affiliations:** Division of Outpatient Pain Clinic, Department of Anesthesiology, The Jikei University School of Medicine, 3-19-18, Nishi-shimbashi, Minato-ku, Tokyo, 105-8471 Japan

**Keywords:** Spontaneous intracranial hypotension, Cerebral venous thrombosis, Epidural blood patch

## Abstract

**Background:**

Spontaneous intracranial hypotension (SIH) is rare but can lead to life-threatening complications including cerebral venous thrombosis (CVT). The concurrence of CVT and SIH raises questions regarding priority.

**Case presentation:**

We present the case of a 52-year-old woman who developed sudden left-sided hemiparesis and generalized tonic-clonic seizures. She experienced progressive orthostatic headaches over the prior 2 weeks. Imaging showed thrombosis in the left transverse and sigmoid sinuses, bilateral subdural hematomas, and a cervicothoracic cerebrospinal fluid leak. Low molecular weight heparin was administered, but it was discontinued 2 days later due to subarachnoid hemorrhage. She was transferred to our hospital where an epidural blood patch was applied immediately, which resulted in complete symptom relief.

**Conclusion:**

CVT is a rare complication of SIH that may result in devastating consequences. Treatment of SIH should be the primary focus. Prompt diagnosis and EBP application can result in a good outcome.

## Background

Spontaneous intracranial hypotension (SIH) is an uncommon entity, resulting from spontaneous cerebrospinal fluid (CSF) leak. Although its primary clinical feature is orthostatic headache, other reported symptoms include cervical pain, dizziness, nausea and vomiting, hearing loss, tinnitus, diplopia, vision loss, and cranial nerve deficits [1–3]. Cranial magnetic resonance imaging (MRI) typically shows pachymeningeal enhancement, subdural collections, downward displacement of the cerebral tonsils, enlargement of cerebral venous structures, and pituitary hyperemia [4]. Given the variety of clinical and radiographic manifestations, SIH often goes unrecognized, and the diagnosis is often delayed.

Although generally benign, SIH can cause life-threatening CVT, which can result in subarachnoid hemorrhage and seizures [5]. Approximately one third of patients with CVT develop focal or generalized seizures prior to diagnosis [6]; these can result in death [7, 8]. Few reports have described sudden seizure as a primary symptom of SIH with CVT[ 9,10]. When a patient presents with both SIH and CVT, a decision regarding priority of treatment is required. First-line treatment for SIH is an application of epidural blood patch (EBP); however, its indication in the setting of concurrent CVT is unknown. Here we report a complex case of SIH and associated CVT and review the literature.

## Case presentation

**Fig. 1:**
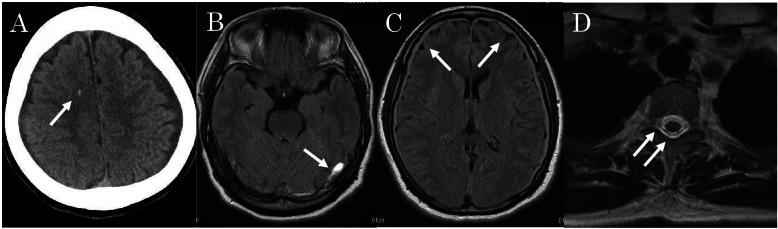
**a** Axial cranial CT showing microbleeds in the right frontal lobe (arrow). **b** Axial cranial MRI showing subdural hematomas (arrow). **c** Axial cranial MRI with contrast showing thrombosis in the left transverse and sigmoid sinuses and narrow lateral ventricle (arrows). **d** Postmyelography CT showing a cervical CSF leak into the epidural space (arrows). CSF, cerebrospinal fluid; CT, computed tomography; MRI, magnetic resonance imaging

An unconscious 52-year-old woman with a history of hypertension presented to the emergency department of an outside hospital after acute onset left-sided hemiparesis and generalized tonic-clonic seizures. She experienced progressive orthostatic headaches during the prior 2 weeks. There was no history of major trauma or lumbar puncture, and she was not taking oral contraceptives or hormonal replacement agents. Emergency cranial computed tomography (CT) showed cerebral microbleeds in the right frontal lobe (Fig. 1a). Cranial MRI with contrast showed thrombosis in the left transverse and sigmoid sinuses, bilateral subdural hematomas, and coarctation of the lateral ventricles (Fig. 1b, c). Spinal MRI with contrast revealed a cervicothoracic CSF leak (Fig. 1d). SIH associated with bilateral subdural hematomas and CVT was diagnosed, and intravenous low molecular weight heparin therapy was initiated; the CVT resolved within a few hours.

Two days later, the patient’s left face and left arm became numb. Cranial CT showed subarachnoid hemorrhage; thus, heparin was discontinued. She then developed left-sided involuntary movements and acute worsening of the hemiparesis the next day. Heparin was initiated as worsening thrombosis was suspected.

**Fig. 2:**
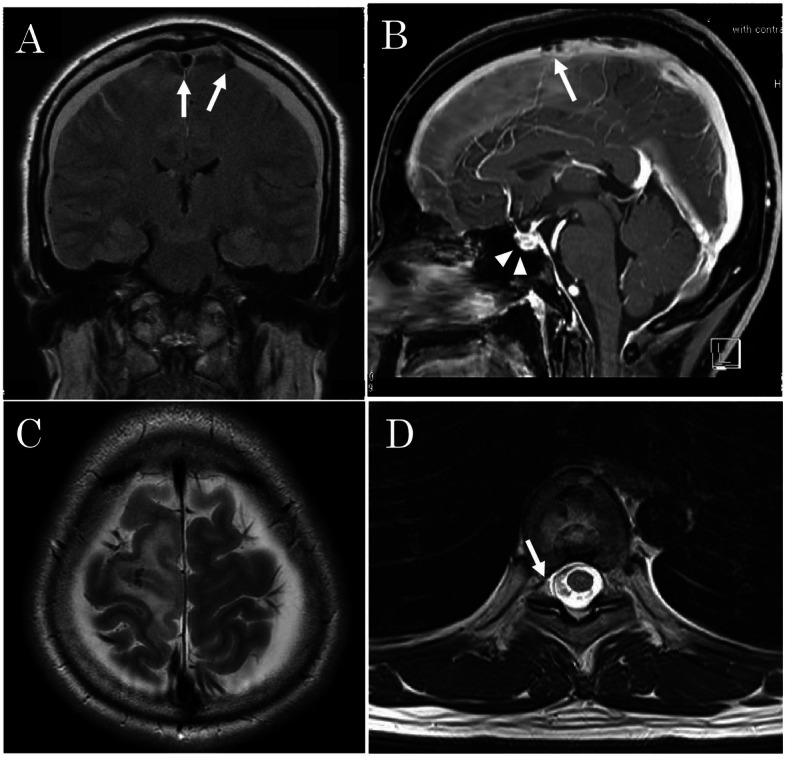
**a** Coronal T1-weighted gadolinium-enhanced MRI showing diffuse pachymeningeal enhancement and cortical vein thrombosis (arrows). **b** Sagittal T1-weighted MRI showing enlargement of the pituitary gland (arrow head) and cortical vein thrombosis (arrow). **c** Axial cranial MRI showing cerebral edema. **d** Spinal MRI showing anterior epidural fluid collection at the level of the T4–T10 spinal canal (arrow). MRI, magnetic resonance imaging

At this point, the patient was transferred to our hospital for further treatment. On arrival, her neurologic examination was unremarkable except for left-sided muscle weakness. Cranial MRI with contrast showed diffuse pachymeningeal enhancement, enlarged pituitary gland, and diffuse cerebral edema (Fig. 2a–c). Although spinal MRI showed an anterior epidural T4-T10 fluid collection, no clear site of CSF leakage was visualized (Fig. 2d). Heparin was discontinued the next day in anticipation of EBP placement.

**Fig. 3:**
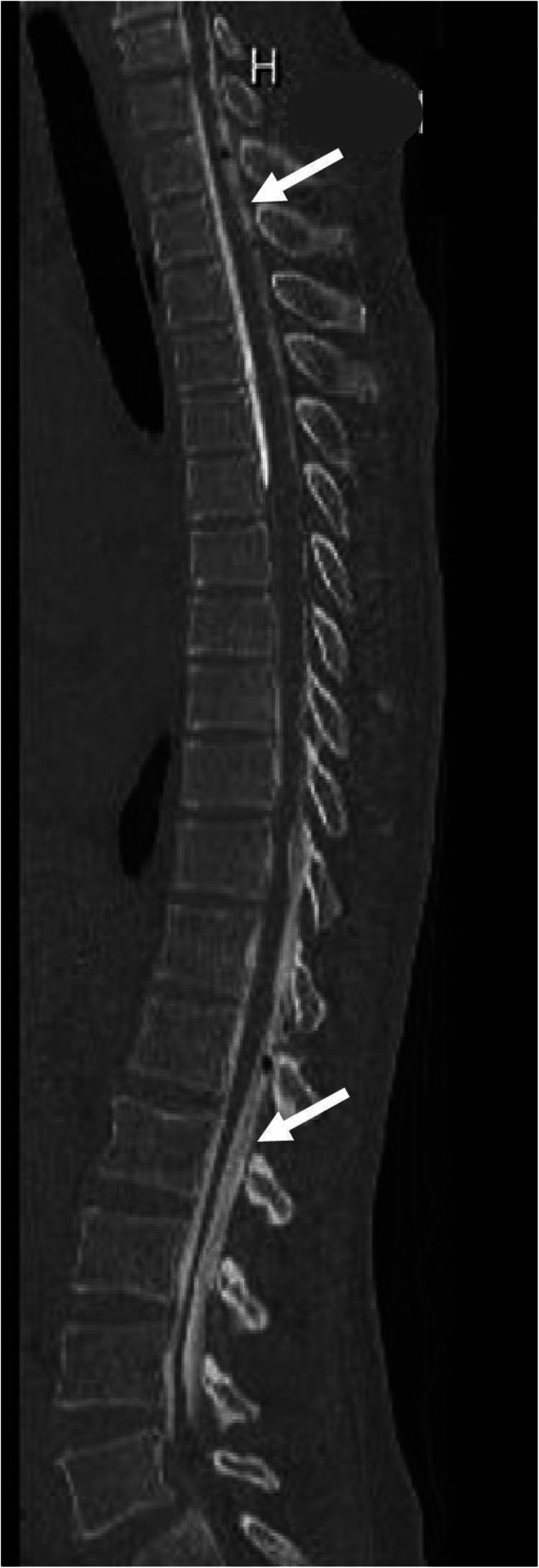
Sagittal cervicothoracic spinal MRI showing hyperintense epidural fluid collection (arrows) indicating sealing of the leakage site. MRI, magnetic resonance imaging

**Fig. 4:**
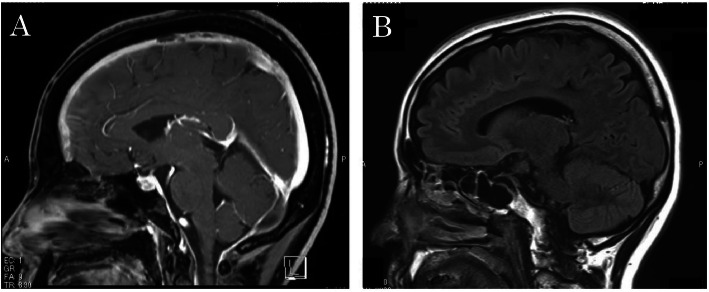
**a** Sagittal T1-weighted gadolinium-enhanced MRI. Baseline imaging shows diffuse pachymeningeal enhancement, sagging of the brain, enlargement of the pituitary gland, and coarctation of the lateral ventricles. **b** Imaging after EBP placement shows resolution of brain sagging, smaller pituitary gland, resolution of pachymeningeal enhancement, and resolution of lateral ventricle narrowing. EBP, epidural blood patch; MRI, magnetic resonance imaging

An EBP was placed via multiple interlaminar injections using a 22-gauge Tuohy needle (Unisis Corp, Tokyo, Japan) under fluoroscopy. After successful loss of resistance, a small volume of contrast medium (Isovist Inj. 240; Bayer, Leverkusen, Germany) was injected to fluoroscopically confirm epidural spread. It was difficult to identify the leakage site; approximately 52 mL of contrast medium and sterile autologous blood mixture (1:4) was injected separately under fluoroscopy at the T1–T2 and T12–L1 levels, which are considered as the most frequent sites. The injection was stopped when the patient began to note tightness in her shoulder. Epidural blood dissemination was subsequently evaluated by spinal CT (Fig. 3). The EBP resulted in improvement of muscle power and headache by the second day after treatment. Intravenous heparin was started 6 h later, and the patient was converted to oral warfarin (6-month course). At the 3- and 6- month follow-up visits, she reported no further headache episodes and had no focal neurologic deficits. Follow-up MRI at 3 months showed recanalization of the thrombosed venous sinuses (Fig. 4).

## Discussion

The association between SIH and CVT was first reported in 2005 [11]. Since then, a small number of cases have been reported. A PubMed database search in April 2019 using the terms “spontaneous intracranial hypotension,” “CSF leak,” and “cerebral venous thrombosis” identified 41 cases [4, 5, 9–36]. Table 1 presents these cases, along with the present patient (iatrogenic cases were excluded). Although the prognosis of SIH and CVT is generally good, life-threatening complications, including seizures and intracranial hemorrhage, can arise. The present patient is the third reported case of diagnosis resulting after a sudden seizure.

**Table 1:** Clinical and radiologic data for 42 patients with CVT

Treatment	Author, year	Age, sex	Headache (orthostatic/change)	Associated symptoms	Imaging (SIH/CVT)	Complications	Complications After treatment	Symptom resolution
AC (*n* = 21)	Berroir et al., 2004 [12]	46 F	+/+	Auditory disturbance	+/+	-	-	Complete
		32 F	+/+	Nausea/vomiting, somnolence	+/+	-	-	Complete
	Sopelana et al., 2004 [13]	56 M	+/−	Nausea/vomiting	+/+	-	-	Complete
	Flemming and Link, 2005 [11]	31 F	+/+	Nausea, eye pain, auditory disturbance	+/+	-	dAVF	Incomplete
	Savoiardo et al., 2006 [14]	31 M	+/−	Nausea/vomiting, blurred vision, auditory disturbance	+/+	-	-	Incomplete
		40 M	−/−	Hemiparesthesia	+/+	Venous infarct	-	Complete
	Richard et al., 2007 [15]	38 M	+/+	-	+/+	SAH, SDH	-	Complete
		60 F	+/−	Hemiplegia, tinnitus	+/+	ICH	-	Complete
	Tan et al., 2008 [16]	46 F	+/+	-	+/+	-	-	NA
		40 M	+/+	Vertigo	+/+	-	-	NA
	Haritanti et al., 2009 [4]	42 M	+/−	Neck stiffness, dizziness, tinnitus, nausea, seizure, horizontal diplopia	+/−	ICH	-	Complete
	Ivanidze et al., 2010 [17]	33 F	+/+	-	+/+	-	-	Complete
	Nardone et al., 2010 [18]	44 M	+/+	Sensory disturbance, visual field defect	+/+	SDH	SDH enlargement	Complete
	Dangra et al., 2011 [19]	35 M	+/+	-	+/+	-	SDH	Complete
	Mao et al., 2011 [20]	34 M	+/+	-	+/+	SDH	SDH enlargement	Complete
	Ferrante et al., 2012 [21]	59 M	−/+	Dizziness, loss of coordination	+/+	-	-	Complete
	Costa et al., 2012 [22]	48 F	+/−	Nausea, transient diplopia, plugged ear, blurred vision	+/+	-	-	Incomplete
	Tian and Pu, 2012 [23]	41 F	+/−	Nausea/vomiting, transient diplopia	+/+	-	-	Complete
	Rice et al., 2013 [24]	75 M	−/−	Status epilepticus	+/+	ICH	Died after further ICH	Death
	Rozen, 2013 [9]	NA	−/−	Seizure	+/+	-	-	NA
	Fujii et al., 2018 [25]	33 F	+/+	Nausea/vomiting, vertigo	+/+	-	-	Complete
EBP (*n* = 7)	Wang et al., 2007 [26]	33 F	+/−	Nausea/vomiting, seizure	+/−	SAH	-	Complete
	Takeuchi et al., 2007 [27]	32 M	+/−	Nausea	+/+	-	-	Complete
	Yoon et al., 2011 [28]	26 M	+/+	-	+/−	-	-	Complete
	Zhang et al., 2018 [29]	34 M	+/+	Tinnitus, seizure, neck stiffness	−/−	-	-	Complete
		43 F	+/+	Nausea, neck stiffness	+/+	SDH	-	Complete
		37 F	+/+	Nausea/vomiting, seizure	+/+	-	-	Complete
		38 M	+/+	Nausea/vomiting, seizure, neck stiffness	+/+	-	-	Complete
AC + EBP (*n* = 13)	Lai et al., 2007 [10]	45 F	+/+	Nausea/vomiting, seizure, numbness	+/+	Venous infarct	-	Complete
	Kataoka et al., 2007 [30]	36 M	+/+	Nausea, auditory disturbance	+/+	-	SDH	Complete
	Albayram et al., 2007 [31]	45 M	+/+	-	+/+	-	-	Incomplete
	Schievink and Maya, 2008 [5]	26 F	+/−	Nausea	+/+	-	-	Complete
		32 M	+/−	Nausea	+/+	SAH	-	Complete
		21 F	+/+	Transient diplopia, seizure	+/−	Pseudo-SAH, venous infarct	-	Complete
	Ade and Moonis, 2013 [32]	54 F	+/+	Nausea/vomiting	+/+	SAH, SDH	-	Complete
	Garcia-Carreira et al., 2014 [33]	29 F	+/−	Photophobia, phonophobia	−/+	-	-	Complete
		54 M	+/+	Dizziness, hemiparesis, hemihypesthesia, numbness	+/−	SAH, ICH	-	Complete
	Sinnaeve et al., 2017 [34]	21 F	+/+	Nausea, photophobia	−/+	-	-	Complete
		30 F	+/+	-	−/+	-	SDH	Complete
	Perry et al., 2018 [35]	43 M	−/+	Seizure, dizziness, nausea, weakness	+/+	-	ICH (hemiplesia, seizure)	Incomplete
	Present study	52 F	+/+	Hemiparesis, seizure	+/+	SAH	-	Complete
None (*n* = 1)	Lan et al., 2007 [36]	36 M	+/+	Nausea/vomiting, seizure	+/+	SDH, ICH	-	NA

*AC* anticoagulation, *CVT* cerebral venous thrombosis, *dAVF* dural arteriovenous fistula, *EBP* epidural blood patch, *ICH* intracerebral hemorrhage, *NA* not available, *SAH* subarachnoid hemorrhage, *SDH* subdural hematoma, *SIH* spontaneous intracranial hypotension

Although CVT affects only approximately 5 persons per million (0.0005%) in the general population [37], the prevalence among patients with SIH increases to 2% [5]. Several SIH pathophysiological mechanisms play a role in CVT development. First, the Monro-Kellie doctrine states that a loss of one component must be compensated by an increase in another in a closed compartment such as the intracranial and spinal subdural space [38]. Therefore, any loss of CSF is replaced by an increase in the most readily expansible component, which is venous blood. Subsequently, venous engorgement results in the slowing of venous blood flow. Given that patients with SIH likely lose more CSF than those undergoing lumbar puncture, the decreased blood flow velocity in patients with SIH is likely greater.

Second, the downward sagging of intracranial structures due to the loss of CSF buoyancy [1] causes traction on cerebral veins and sinuses [4], which leads to venous flow turbulence or stasis. Third, the loss of CSF decreases CSF absorption into the cerebral venous sinuses, leading to an increase in cerebral venous blood viscosity [39]. Therefore, the occurrence of this rare condition is unpredictable and results in outcomes ranging from complete remission to severe disability or death.

Both SIH and CVT are frequently misdiagnosed, which results in treatment delay and increased risk of complications. The present case highlights the importance of prompt imaging and aggressive treatment. However, in our literature review (including this case), many patients received an incorrect initial diagnosis despite MRI results evidencing SIH in 38 patients (90%) and CVT in 36 patients (86%) (Table 1). In addition, 28 patients (67%) reported a small or sudden change in their headache pattern. None of the patients had clinical or radiographic evidence of CVT preceding the development of SIH. Twelve cases (29%) presented with seizures (Table 1); however, only 3 (7%) had seizures as a primary symptom (present patient, [10, 11]). We found no features that predicted seizures other than headache, which is almost always the primary symptom of SIH. Therefore, it is important to closely monitor patients for any change in headache pattern.

In most reports, CVT was treated with anticoagulation (Table 1). The current consensus regarding CVT treatment with anticoagulation is based on 2 small randomized studies that concluded that it is safe and is associated with decreased risk of death and disability [40, 41]. However, anticoagulation theoretically increases the risk of bleeding and subdural hematoma (SDH) development, which is a concern in patients with SIH. Although the development or worsening of intracranial hemorrhage may not be entirely attributable to anticoagulation, 5 patients (12%) in our literature review developed SDH or SDH enlargement after treatment, and 2 (5%) developed intracranial hemorrhage or worsening of intracranial hemorrhage (Table 1); the patient with worsened intracranial hemorrhage died. Consequently, the benefit of anticoagulation should always be weighed against the risk of SDH, especially when subdural fluid collections are present [20].

The mainstay of SIH treatment is EBP placement; however, its effect on venous thrombosis development and recanalization is unknown. We hypothesized that the primary treatment focus should be SIH, given that CVT development is closely related to SIH-induced pathophysiologic changes in the brain. Thus, we prioritized EBP placement, even though the present patient had anticoagulation initiated at another hospital. In our literature review (including the present case), of the 34 patients who received anticoagulation therapy, 14 of 21 (67%) who did not receive an EBP had a complete neurologic recovery; in contrast, 11 of 13 (85%) who received an EBP recovered completely. Seven patients underwent EBP without anticoagulation, and all completely recovered. These data suggest that EBP application might be warranted for consideration as first-line therapy.

There are several limitations of this literature review. It was retrospective in nature and examined only 42 cases due to the low prevalence of SIH and CVT. Moreover, anticoagulation therapy and EBP application were not performed in the same manner across studies, although treatments likely improved over time.

## Conclusion

We report a complex case of SIH associated with CVT, a rare complication with potential severe neurologic and life-threatening consequences. It is difficult to prevent sudden seizures before diagnosis; if seizures occur, immediate advanced diagnostic imaging should be performed along with supportive neurologic care. We believe that the primary focus of treatment should be directed at SIH, given that the underlying CVT cause is closely related to the pathophysiologic changes that occur after SIH. Prompt diagnosis and treatment with EBP application can result in a good outcome, as in the present case. Although the prevalence of SIH with CVT is low, rendering case-controlled studies impractical, additional studies are needed to determine an algorithm for early diagnosis and treatment.

## Data Availability

Not applicable.

## Abbreviations

CSF: Cerebrospinal fluid
CT: Computed tomography
CVT: Cerebral venous thrombosis
EBP: Epidural blood patch
MRI: Magnetic resonance imaging
SDH: Subdural hematoma
SIH: Spontaneous intracranial hypotension
